# Exploratory clinical trial on the safety and capability of dMD-001 in lumbar disc herniation: Study protocol for a first-in-human pilot study

**DOI:** 10.1016/j.conctc.2021.100805

**Published:** 2021-06-29

**Authors:** Katsuhisa Yamada, Maeda Kenichiro, Yoichi M. Ito, Fujio Inage, Toshiyuki Isoe, Nozomi Yokota, Osamu Sugita, Norihiro Sato, Khin Khin Tha, Norimasa Iwasaki, Teruyo Arato, Hideki Sudo

**Affiliations:** a Department of Orthopaedic Surgery, Hokkaido University Hospital, N14W5, Sapporo, Japan; b Department of Orthopaedic Surgery, Faculty of Medicine, and Graduate School of Medicine, Hokkaido University, N15W7, Sapporo, Hokkaido, 060-8638, Japan; c Clinical Research and Medical Innovation Center, Hokkaido University Hospital, N14W5, Sapporo, Japan; d Department of Biostatistics, Hokkaido University Graduate School of Medicine, N14W5, Sapporo, Hokkaido, Japan; e Department of Diagnostic Imaging, Hokkaido University Hospital, N14W5, Sapporo, Hokkaido, Japan; f Department of Advanced Medicine for Spine and Spinal Cord Disorders, Faculty of Medicine and Graduate School of Medicine, Hokkaido University, Japan

**Keywords:** Intervertebral disc herniation, Ultra-purified alginate gel, First-in-human pilot study

## Abstract

Herniated nucleus pulposus (NP), one of the most common diseases of the spine, is surgically treated by removing the sequestered NP. However, intervertebral disc (IVD) defects may remain after discectomy, leading to inadequate tissue healing and predisposing patients to IVD degeneration. An acellular, bioresorbable, ultra-purified alginate (UPAL) gel (dMD-001) implantation system can be used to fill any IVD defects in order to prevent IVD degeneration after discectomy. This first-in-human pilot study aims to determine the feasibility, safety, and perceived patient response to a combined treatment involving discectomy and UPAL gel implantation for herniated NP. We designed a one-arm, double-centre, open-label, pilot trial. The study started in November 2018 and will run until a sample of 40 suitable participants is established. Patients aged 20–49 years, diagnosed with isolated lumbar IVD herniation and scheduled for discectomy represent suitable candidates. All eligible participants who provide informed consent undergo standard discectomy followed by UPAL gel implantation. The primary outcomes of the trial will be the feasibility and safety of the procedure. Secondary outcomes will include self-assessed clinical scores and magnetic resonance imaging-based measures of morphological and compositional quality of the IVD tissue. Initial outcomes will be published at 24 weeks. Analysis of feasibility and safety will be performed using descriptive statistics. Both intention-to-treat and per-protocol analyses of treatment trends of effectiveness will be conducted.

## 1 Introduction

Herniated nucleus pulposus (NP), one of the most common diseases of the spine, is surgically treated by removing the sequestered NP. Discectomy is performed to remove intervertebral disc (IVD) material impinging on the nerve root, thereby alleviating pain. However, defects in the IVD may remain after discectomy, leading to inadequate tissue healing and predisposing patients to IVD degeneration. In addition, this open defect can serve as a point of egress for re-herniation, potentially resulting in recurrent symptoms and the need for further surgery [1]. To prevent re-herniation, surgeons are inclined to remove as much IVD material as possible, sometimes removing even material not displaced from within the annulus fibrosus (AF) [1]. While this practice may reduce re-herniation risk, it reportedly accelerates IVD degeneration, resulting in worse clinical outcomes and long-term back pain [[1], [2], [3]].

The regeneration capacity of IVDs is limited due to poor nutritional supply, low oxygen tension, acidic pH, low cell density, and cell viability and genetic predisposition [4,5]. Efforts to address this challenge have focused on biological repair of the whole IVD with NP and AF composite tissues. This is achieved using tissue engineering and regenerative medicine in combination with classical surgical procedures [[5], [6], [7]].

Although several basic research studies have demonstrated that alginate supports the reparative capacity of IVD [7,8], both naturally occurring and commercial-grade alginates contain mitogenic or cytotoxic impurities, which may induce foreign body reactions [9]. To explore the reparative capacity of alginate for tissue engineering in clinical applications while minimizing the risk of adverse effects, we previously developed a highly pure, biocompatible alginate with reduced endotoxicity (<50 EU/mL) [10]. An acellular bioresorbable ultra-purified alginate (UPAL) gel (dMD-001) implantation system was also developed for filling the IVD defect after discectomy, thus stimulating the ingrowth of reparative cells, promoting NP tissue formation, and preventing IVD degeneration. The feasibility of this treatment option, with formation of repair tissue and improvement of IVD water content, has been demonstrated in an animal model [11]. Thus, the next step was to design a first-in-human pilot study aiming to determine the feasibility, safety, and perceived patient response to a combined treatment involving discectomy and UPAL gel implantation for herniated NP. Should such a study indicate that the treatment may be effective, the collected data will be used to conduct a sample size calculation for a fully-powered trial.

## 2 Methods

### 2.1 Ethics

This study was designed in accordance with the Declaration of Helsinki and was approved by the ethics committees of Hokkaido University Hospital (approval number: H30-10) and Eniwa Hospital (approval number: dMD001-H1), as well as by the Japanese Pharmaceuticals and Medical Devices Agency (Japan). The trial will follow the Good Clinical Practice guidelines issued by the Japanese Ministry of Health, Labour and Welfare. Written informed consent is obtained from all patients before enrolment.

### 2.2 Trial design

**Fig. 1:**
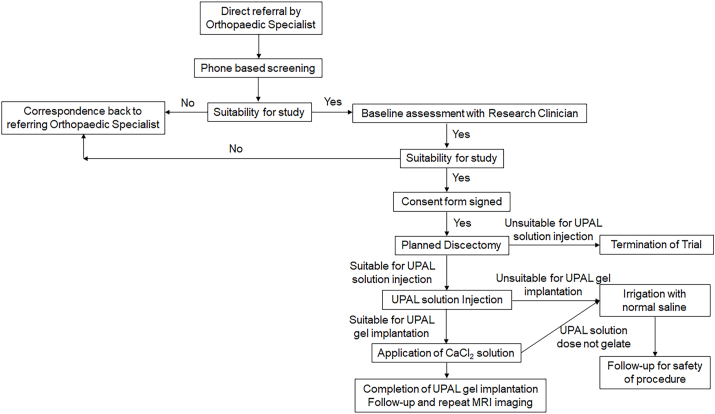
Study flow chart. MRI: Magnetic resonance imaging, UPAL: Ultra-purified alginate.

This first-in-human study is being conducted as a double-centre, open-label, pilot trial. An overview of the process of the trial is presented in Fig. 1 and Table 1. Protocol is version 1.0 and the protocol was fixed on July 5, 2018. The study started in November 2018 and recruitment of patients will end in September 2020 or will run until a sample of 40 suitable participants is established. And the last patient has been recruited in September 2020.

**Table 1:** **Schedule of enrolment, intervention, assessment, and follow-up**.

	Study period					
	Enrolment	Operation	Post-operation			
Time point (week)	−1	0	1	4	12	24
**Enrolment**						
Eligibility screening	X	X				
Obtaining informed consent	X					
**Assessment**						
Recording demographic characteristics	X					
Physical examination/vital signs evaluation	X		X	X	X	X
Allergy test for sodium alginate (skin prick testing)	X					
Laboratory tests	X		X	X	X	X
Visual analog scale scoring for back pain and leg pain	X		X	X	X	X
Finger-to-floor distance test	X		X	X	X	X
Straight-leg-raising test	X		X	X	X	X
Modified Schober's test	X		X	X	X	X
Japanese Orthopaedic Association (JOA) scoring	X		X	X	X	X
36-item Short Form Health Survey	X		X	X	X	X
Oswestry Disability Index evaluation	X		X	X	X	X
Rolland-Morris disability questionnaire	X		X	X	X	X
JOA Back Pain Evaluation Questionnaire	X		X	X	X	X
Radiographic evaluation	X	X				
Magnetic resonance imaging evaluation	X					X
Reporting adverse events						
Recording medical/drug use history						

### 2.3 Participants

Both pre-operative and surgical inclusion/exclusion criteria have been established for this trial. Specifically, the study will recruit participants with symptomatic herniated NP unresponsive to nonoperative care and considered candidates for nerve decompression and surgical excision of the herniated lumbar IVD fragments [1]. Clinical signs and symptoms, as well as magnetic resonance imaging (MRI) findings must corroborate the diagnosis of lumbar IVD herniation. Patients with previous lumbar surgery will not be considered for this trial. Furthermore, only patients who receive single-level discectomy will be considered eligible to receive UPAL gel implantation.

Upon completion of standard discectomy, the treating surgeon will assess the feasibility of AF re-approximation and make the final decision whether the patient will receive UPAL gel implantation and be officially enrolled in the study [1]. The complete inclusion/exclusion criteria are as follows [1].

Inclusion criteria.

i. Candidate for lumbar discectomy.
ii. Radiographic findings corroborating symptoms of IVD herniation.
iii. Condition unresponsive to 6 consecutive weeks of therapy or experiencing acute/uncontrolled leg pain, defined as a score >80 on the 100-mm visual analog scale (VAS), in which higher scores representing worse pain correspond to worse pain.
iv. Single-level lumbar IVD herniation.
v. Persistent and predominant leg pain (score >40 on the 100-mm VAS)
vi. Age between 20 and 49 years (inclusively).
vii. Willingness to provide written informed consent, fill in all necessary questionnaires, and return for follow-up.

Exclusion criteria.

i. Previous surgery involving a lumbar level.
ii. Prior or planned spinal fusion involving a lumbar level.
iii. Local kyphosis involving the affected disc level, evident on plain radiography of the lumbar spine in the flexion, neutral or extension position.
iv. Spondylolisthesis or retrolisthesis above grade 1 at the affected level.
v. Cauda equina syndrome.
vi. Acute local or systemic infection.
vii. Active malignancy or other similar comorbidities.
viii. Current drug or alcohol dependency.
ix. Current significant emotional disturbance.
x. Current fracture, tumour, and/or deformity of the lumbar spine.
xi. Current or planned pregnancy.
xii. Currently enrolled in other research that could confound the results of the present trial.
xiii. Presence of a metal implant or any other contraindication to MRI.
xiv. Allergy to sodium alginate revealed upon skin prick testing.
xv. Any other reason judged by an investigator or clinical trial doctor to render the candidate unsuitable for this clinical trial.

Eligible candidates will be treated at the Hokkaido University Hospital in Sapporo, Japan, or at Eniwa Hospital in Eniwa, Japan, depending on where the referring orthopaedic surgeons have formal accreditation. The treatment will include discectomy followed by UPAL gel implantation. The discectomy procedure is part of the accepted standard treatment of each participant but is not part of the present study. The discectomies will be performed by the referring surgeons and will not be performed by a single surgeon.

### 2.4 Recruitment

Candidates are being recruited from local orthopaedic hospitals. Patients with symptomatic herniated NP indicated for surgical treatment are invited to fill in a screening questionnaire to determine their eligibility. Patients who meet the inclusion criteria are contacted by one of the investigators to confirm their willingness to participate in the trial and arrange a baseline assessment, during which written informed consent is also obtained. Patients selected for the study may continue to use their prescribed medication for the duration of the trial. The type of medication and dosage used is recorded at the baseline assessment.

### 2.5 Interventions

#### 2.5.1 Discectomy

All participants will undergo macroscopic discectomy for lumbar IVD herniation. These open procedures will be conducted using standard or tubular retractors, with or without the use of an operating microscope or loupes [1]. Manual, nonautomated methods for herniated IVD removal will be used exclusively, and no percutaneous methods will be applied [1]. To standardize the fenestration site for UPAL gel implantation, the procedures will involve an AF incision of 5 mm × 5 mm using a no. 15 surgical scalpel blade. In patients with transligamentous extrusion-type or sequestration-type herniated disc, the AF opening will be measured and reported. After the AF incision, we aim to remove as much disc material as possible. It will not be a limited discectomy. The technique used for discectomy will be at the discretion of the treating surgeon [1]. Whilst the surgeons’ preference and skill may confound the outcomes, this represents an acceptable limitation that has been purposefully allowed in order to facilitate recruitment of participants from across a wider orthopaedic community [12].

Stopping criteria before UPAL gel implantation.

i. The AF incision for discectomy exceeds 5 mm × 5 mm (i.e., the diameter of the incision exceeds 5 mm).
ii. The treating surgeon judges that AF re-approximation is not feasible or there is some other reason rendering the patient unsuitable for UPAL gel implantation such as a spinal fluid leak due to an incidental tear of the dura mater.
iii. The volume of sterile, clinical-grade, normal saline delivered into the IVD defect exceeds 2.0 mL, indicating rupture of the AF.

If either of the above criteria for termination is met, no UPAL gel implantation will be conducted after the standard discectomy, and the patient will be excluded from the trial. The reason for stopping the UPAL gel implantation will be recorded.

#### 2.5.2 UPAL gel (dMD-001) implantation

UPAL gel (dMD-001) is manufactured by quality management system and is provided by Mochida Pharma Co. Ltd (Mochida Pharma Co. Ltd., Tokyo, Japan). Following standard discectomy, the treating surgeon will evaluate the feasibility of AF re-approximation. If the evaluation is positive, a board-certified orthopaedic specialist will inject the UPAL gel into the IVD. Specifically, up to 2 mL of UPAL solution will be delivered into the IVD defect using a syringe, and 10 mL of 0.1 mol/L CaCl_2_ solution will then be applied to the surface of the UPAL solution to gelate the alginate. At 5 min after application of CaCl_2_ solution, the surgical site will be irrigated with a full-dose of sterile, clinical-grade, normal saline. All patients will receive standard postoperative care will be implemented. Braces after surgery will not be used.

Stopping criteria during UPAL gel implantation.

i. The volume of UPAL solution delivered into the IVD defect exceeds 2.0 mL.
ii. The UPAL solution does not gelate upon treatment with CaCl_2_.
iii. Any other reason judged by the treating surgeon to render the participant unsuitable for the trial at this time such as an allergic reaction.

If either of the above terminating criteria is met, the surgical site will be irrigated with a full-dose of sterile, clinical-grade, normal saline. Assessment of safety after injection of the UPAL solution will be performed for up to 24 weeks. The reason for stopping the UPAL gel implantation will be recorded.

### 2.6 Measures

#### 2.6.1 Primary outcome measurement

The primary outcomes of this trial are the feasibility and safety of the procedure. Feasibility is defined based on the quality-control release criteria for the clinical use of UPAL as a biomaterial, which refer to the suitability of the biomaterial for intraoperative manipulation and implantation into the IVD defect [13]. Safety is defined in terms of the incidence of serious adverse events and serious adverse reactions, according to the Pharmaceutical and Medical Device Act currently in effect in Japan [13]. Any adverse events, either local (e.g., infection, haematoma) or systemic (e.g., fever, allergic reaction), will be handled according to the Japanese guidelines for Good Clinical Practice. The following serious adverse events will be recorded [1]:

i. Abdominal pain
ii. Back pain
iii. Back and leg pain
iv. Cancer
v. Death
vi. Dural tear
vii. Heat attack/cardiac arrest
viii. Haematoma/seroma
ix. Hip pain
x. Leg pain
xi. Neck/cervical pain
xii. Pneumonia/atelectasis
xiii. Spinal fluid leak
xiv. Wound infection
xv. Local or systemic fever
xvi. Abnormal blood coagulation
xvii. Recurrence of herniation

Recurrent herniation is defined as re-herniation at the same vertebral level. A group of non-investigator physicians will independently adjudicate the primary cause for any secondary surgery involving the same vertebral level. Re-herniation will be considered recurrent if all extruded NP material is removed during the initial surgery and a new offending fragment, including the gel, is identified, or if there is evidence that not all extruded NP material had been removed during the initial surgery and residual material remains [1].

#### 2.6.2 Secondary outcome measurement

The secondary outcomes of this trial are: (i) physical function scores; (ii) self-reported patient satisfaction at 1 day before surgery (baseline) and at 1, 4, 12, and 24 weeks after surgery; and (iii) morphological and compositional quality of the IVD tissue evaluated on MRI before surgery (baseline) and at 24 weeks after surgery.

#### 2.6.3 Physical examination to evaluate physical function

The finger-to-floor distance will be measured as the shortest distance (in cm) between the floor and fingertips as the patient stands and bends down from the waist with arms stretched, without bending the knees [14,15]. The straight-leg-raising test will be conducted to measure the maximum angle (in degrees) between a horizontal surface and the lower extremity as the patient raises the leg by flexing the hip, with the knee passively extended [14]. The range of motion of the lumbar spine will be measured using the Modified Schober's test; specifically, three marks (one at the lumbosacral junction, one at the spinous process lying 10 cm above the first mark, and one at the spinous process lying 5 cm below the first mark) will be made with the patient in the standing position, and the range of motion will be measured as the distance (in cm) between the inferior and superior marks at full hip extension minus the distance at full hip flexion [16]. The Japanese Orthopaedic Association (JOA) score will be used to evaluate functional status and the severity of clinical symptoms associated with lumbar disc herniation; the JOA score ranges from 0 to 29, with higher scores indicating better functional status [16].

#### 2.6.4 Self-reported questionnaires for evaluating pain and health-related quality of life

A 100-point VAS (0–100 mm, with higher scores representing worse pain) will be used to assess leg pain on both the worst-affected and least-affected side, as well as back pain. The preoperative VAS score for the worst-affected leg will be considered as the reference for postoperative comparisons [1]. Health-related quality of life will be assessed using the 36-item Short Form Health Survey (0–100, with higher scores representing better quality of life) [17]. Overall functional outcome will be assessed using the Oswestry Disability Index (0–100, with higher scores indicating more severe pain-related disability) [18]. Low-back pain-specific quality of life will be evaluated using the Roland-Morris disability questionnaire (0–24, with higher scores representing worse quality of life) [19]. Multidimensional evaluation of health status, including dysfunctions, disabilities, and psychosocial problems associated with the lumbar spine disorder will be assessed using the JOA Back Pain Evaluation Questionnaire (0–100, with higher scores representing better health status) [20]. The use of medication during the week preceding each follow-up visit will be recorded at 1, 4, 12, and 24 weeks postoperatively. Medication intake is considered a useful surrogate for outcome measures such as pain and may represent a confounding factor or cointervention [12].

#### 2.6.5 MRI protocol for evaluating the IVD tissue

MRI scans obtained at enrolment and 24 weeks postoperatively will be assessed by a trained investigator. All MRI examinations will be performed with a 3.0-T scanner. First, sagittal conventional T2-weighted images will be obtained using a turbo spin-echo sequence. Next, sagittal T1ρ-, T2*-, and diffusion-weighted images will be obtained using a 3D gradient-echo or an echo planar-imaging pulse sequence [[21], [22], [23]].

For each participant, two board-certified orthopaedic specialists with over 10 years of experience will independently assess the treated lumbar IVD on mid-sagittal T2-weighted images according to the Pfirrmann grading system [24], as described below:

i. Grade I - homogeneous shape, bright hyperintense (white), normal IVD height, clearly distinguishable AF and NP.
ii. Grade II - nonhomogeneous shape with or without horizontal grey bands, hyperintense (white) signal, normal IVD height, clearly distinguishable AF and NP.
iii. Grade III - nonhomogeneous shape, intermediate (grey) signal intensity, normal or slightly decreased IVD height, AF and NP not clearly distinguishable.
iv. Grade IV - nonhomogeneous shape, hypointense (dark grey) signal, normal to moderate decrease in IVD height, completely indistinguishable AF and NP.
v. Grade V - similar to grade IV, but with collapsed IVD space.

The intervertebral disc height index will be obtained as the ratio between the IVD height and the proximal vertebral body height, both measured for the middle portion, on mid-sagittal T2-weighted images [25,26].

After Pfirrmann grading of IVD degeneration is carried out based on mid-sagittal T2-weighted images, T1ρ, T2* and apparent diffusion coefficient values of NP and AF will be assessed. The 3D gradient-echo images with different spin lock and echo times will be fitted on a pixel-by-pixel basis to generate a T1ρ and T2* relaxation time maps, respectively [21]. Regions of interest involving the NP, anterior AF, and posterior AF will be set manually on the T1ρ and T2* maps with reference to the T2 images. The values obtained for the anterior and posterior AF will be averaged to determine a single value representative of the AF [21]. The T1ρ, T2*, and apparent diffusion coefficient values of the NP and AF will be assessed as secondary outcomes. All measurements will be performed twice by a single observer at an interval of 4 weeks.

### 2.7 Sample size

This is a first-in-human pilot study designed to generate data that can be used to inform a future large randomised controlled trial should the intervention appear feasible and safe, and show trends of effectiveness. To date, no data has been published on the quantitative interactions of a biomaterial with the IVD tissue and how such interactions might affect the IVD space after discectomy. Therefore, we could not conduct a priori sample size calculations for this pilot study because the effect size cannot be estimated at this time. The study aims to recruit 40 participants [12] because we expect that such a sample size will be achieved within a reasonable time based on the study recruitment rates within our community. Moreover, a sample size of 40 participants is considered sufficient for an exploratory clinical trial because it allows to detect with 95% probability differences in one or more adverse events that exhibit an incidence ratio of 7.5%. We aim to evaluate key trial parameters such as preliminary indications of effectiveness and to advise of our calculation of a sample size for powering a full trial in the future [27]. Published guidelines recommend that pilot studies be undertaken to allow trial protocols to be tested under study conditions prior to evaluation in a full randomised controlled trial [12,28]. Thus, the results of this limited phase II trial will also assist in determining the relevance and possible benefits of performing a significantly larger phase III randomised controlled trial [12].

### 2.8 Feasibility and safety analysis

Data regarding feasibility and safety will be analysed using descriptive statistics [27]. The percentage of participants who (i) meet the inclusion criteria, (ii) agree to participate in this study, (iii) complete the intervention, and (iv) attend the follow-up assessment will be calculated. Feasibility will be described in terms of the (i) number of follow-up sessions attended by each participant, (ii) number of drop-outs, and (iii) proportion of participants recruited from the total number screened [27]. Additionally, feasibility will be evaluated as the rate of completion of UPAL gel implantation, which will be judged according to the adequacy of gelation of the surface of the injected UPAL solution after application of CaCl_2_ solution. The rate of completion of UPAL gel implantation will be assessed as the number of patients with adequate gelation divided by the number of patients who undergo discectomy followed by UPAL solution injection. Safety will be assessed as the rate of adverse events and reactions.

### 2.9 Statistical analysis

Statistical analyses will be conducted using SPSS version 22 (IBM Corp., Armonk, NY, USA). Data will be expressed as mean number ± standard deviation and frequency (percentage), as appropriate. Clinical scores and radiologic properties of the IVD at different time points during follow-up will be assessed using the paired *t*-test or Wilcoxon signed rank test, according to the normality of the population, which will be determined using D'Agostino-Pearson omnibus tests [13]. A two-tailed probability level of 0.05 will be considered significant. Continuous data will be analysed using linear mixed models, as such models are robust in longitudinal data analysis and account for correlations associated with repeated measurements [12]. As recommended by the revised Consolidated Standards of Reporting Trials statement, the mixed models will adjust for the baseline score of the outcome of interest [12,29]. Original data will be analysed using the Mann-Whitney *U* test. The risk ratio, risk difference, and number needed to treat will be calculated, together with 95% confidence intervals [12,29,30]. Statistical significance will be determined by χ^2^ analysis.

## 3 Discussion

The main aim of this study is to examine the feasibility and safety of the combined treatment involving discectomy and UPAL gel implantation for herniated NP. In addition, measures of physical function scores, self-reported questionnaires for evaluating pain and health-related quality of life and MRI analysis for evaluating the IVD are performed to investigate perceived patient response to the procedure as secondary outcome measurement. Although surgeon can decide whether alginate implantation is feasible, resulting in a possible selection bias, the reason for stopping the implantation will be recorded and the feasibility will be analysed. This is a first-in-human pilot study designed to generate data that can be used to inform a future, large, randomised controlled trial should the intervention appear feasible and safe, and show trends of effectiveness. After the completion of this clinical trial, an observational study will be conducted as a historical control study in patients with lumbar disc herniation who have undergone discectomy only, using the same evaluation protocol as in this clinical trial.

## Author contribution

HS is the principal investigator in this study. HS led the design of this pilot study and filed the funding application. KY, KM, FI, TI, NY, OS, NS, KKT, NI, and TA have all contributed to the design of the study and to drafting the study protocol. HS and KY drafted the manuscript. MYI provided statistical expertise to support the study methodology. KKT is conducting MRI analysis. All authors approved the final version of the manuscript.

## Funding

This study was supported by the 10.13039/100009619Japan Agency for Medical Research and Development [20lm0203045h0003].

## Trial registration

The protocol of this study has been registered with the University hospital Medical Information Network Clinical Trials Registry (UMIN-CTR) (trial registration number: UMIN000034227. Registered 21 September 2018).

## Declaration of interests

The authors declare that they have no known competing financial interests or personal relationships that could have appeared to influence the work reported in this paper.
